# Response and Deterioration Mechanism of Bitumen under Acid Rain Erosion

**DOI:** 10.3390/ma14174911

**Published:** 2021-08-29

**Authors:** Xuemei Zhang, Inge Hoff, Rabbira Garba Saba

**Affiliations:** 1 Department of Civil and Environmental Engineering, Norwegian University of Science and Technology, Høgskoleringen 7A, 7491 Trondheim, Norway; inge.hoff@ntnu.no; 2 Norwegian Public Roads Administration, Abels Gate 5, 7030 Trondheim, Norway; rabbira.saba@vegvesen.no

**Keywords:** bitumen, acid rain, erosion, hydrogen ion, morphology, physical property, chemical property, rheological property, deterioration mechanism

## Abstract

Acid rain as an important environmental issue has a negative impact on bitumen performance, thereby shortening the service life of asphalt pavements. Thus, this research aims to investigate the response of bitumen to acid rain and its deterioration mechanism. For this purpose, the simulated acid rain was prepared to erode neat bitumen and short-term aged bitumen. The hydrogen ion concentration of the acid rain, and the morphological, physical, chemical, and rheological properties of the bitumen were evaluated by means of a pH meter, scanning electron microscopy, physical tests, Fourier transform infrared radiation with attenuated total reflectance, and dynamic shear rheometer. The results showed that bitumen properties were severely affected by acid rain, and the changes in bitumen properties were highly related to the erosion time, leading to a reduction in pH value by 0.2 of residual acid rain, rougher bitumen surface, and stiffer bitumen with more oxygen-containing functional groups and fewer carbonyl acid groups (around 10% decrement) after 90 days erosion. These changes contributed to two deterioration mechanisms: oxidation and dissolution of carbonyl acid. Oxidation and dissolution are, respectively, the dominant actions for neat bitumen and aged bitumen during the erosion process, which eventually leads to various responses to acid rain.

## 1. Introduction

Rapid industrial and economic development causes negative changes in the environment, including acid rainfall. Acid rain consisting of sulfuric acid (H_2_SO_4_) and nitric acid (HNO_3_) has adverse effects on asphalt pavements, and H_2_SO_4_ and HNO_3_ are the results of sulphur dioxides (SO_2_) and nitrogen oxides (NO_x_) reacting with water in the air [1]. SO_2_ and NO_x_ are normally released from industrial processes in most regions, such as the north-eastern section of the United States, south-eastern section of Canada, Central Europe, China, and India [2]. Thus, the content of sulfuric acid and nitric acid in acid rain is determined by the emission of SO_2_ and NO_x_ into the atmosphere. According to the latest statistical data collected from Europe [3], NO_x_ emission is almost twice the SO_2_ emission. Therefore, the simulated acid rain in this research comprised sulfuric acid (H_2_SO_4_) and nitric acid (HNO_3_) with the proportion of 1:2. Acid rain is defined as precipitation with a pH value lower than 5.6 [4]. In Northern Europe, the pH value of the precipitation is normally between 4 and 6 [5]. The lowest pH value of 4 was selected for simulating acid rain in this research work. Moreover, the rainfall varies from region to region due to climatic and topographic factors. In this study, 7, 28, and 90 days were chosen as erosion periods to simulate the short-term, mid-term, and long-term acid rain erosion. The different erosion days relate to the service life of pavement in different areas. For example, 7 days can simulate the total erosion time of the roads in arid areas under acid rain conditions, while 90 days for the roads in rainy areas.

As the most common pavement type, asphalt pavements are widely applied worldwide. However, acid rain as an acid solvent will lead to deterioration of asphalt pavements, thereby shortening the service life of the asphalt pavement [6]. Some studies have investigated the effect of acid rain on asphalt mixtures. Due to the erosion caused by acid rain, the physical properties of asphalt mixtures were influenced. For example, increased mass loss and air voids were found after acid rain erosion [7]. Meanwhile, the mechanical properties of asphalt mixtures were also affected by acid rain. For example, the indirect tensile strength, elastic modulus, compressive strength, failure strain, and deformation resistance of asphalt mixture decrease after acid rain erosion [8,9]. Shu found that the acid rain could inhibit the healing ability of asphalt mixtures, inducing bad mechanical performance [10]. It is found that the greater the acidity of an acid rain solution, the more apparent the effect on the mechanical properties of the asphalt mixtures [11]. The changes in physical and mechanical properties of asphalt mixtures finally lead to the stripping of the asphalt layer, which is one of the most significant distresses for asphalt pavements [12]. The detrimental mechanism of acid rain on asphalt mixtures is connected to the adhesion between bitumen and aggregates, which could be significantly reduced after acid rain erosion. Acid rain erosion causes some pieces of the bituminous binder to be exfoliated, which in turn leads to deterioration in mechanical properties of asphalt mixtures [13,14]. Few studies that investigated the influence of acid rain on bitumen have mainly focused on the chemical and adhesion properties of bitumen as influenced by acid rain. For instance, the light components within bitumen decreased, asphaltenes content increased [15,16]; acid makes bitumen more hydrophilic so that the bitumen is vulnerable to acid rain erosion [17].

Therefore, most researchers concentrated more on the influence of acid rain on asphalt mixture performance or the coupling effect of several factors on bitumen performance [18,19,20]. However, a few studies explored the effect and deterioration mechanism of bitumen and acid rain. This study aims to evaluate the response of bitumen to acid rain and study the deterioration mechanism between bitumen and acid rain. For this purpose, simulated acid rain consisting of sulfuric acid and nitric acid with pH 4 was prepared to erode neat bitumen and short-term aged bitumen for 7, 28, and 90 days, respectively. After the erosion process, the hydrogen ion concentration of the acid rain, and the morphology, physical properties, chemical structure, and rheological properties of the bitumen were analysed by conducting pH meter, scanning electron microscopy, physical tests, Fourier transform infrared radiation spectroscopy with attenuated total reflectance, and dynamic shear rheometer tests, as well as the development of bitumen over erosion time. The findings obtained from this research provide a reference for understanding the causes of asphalt pavement distresses caused by acid rain and drawing up subsequent plans for mitigating damages.

## 2. Materials and Methods

### 2.1. Materials and Materials Preparation

#### 2.1.1. Bitumen

The pen 70/100 bitumen is the most widely used bitumen grade in Norway. This bitumen is obtained from the Veidekke company. Two states of the bitumen were studied in this research: neat bitumen and aged bitumen after thin film oven test (TFOT) according to EN 12607-2:2014 [21]. The physical and chemical properties of the neat bitumen and TFOT aged bitumen are displayed in Table 1.

#### 2.1.2. Simulated Acid Rain

The process of simulating acid rain is described as follows. A blend of concentrated acids was obtained by mixing 1 g sulfuric acid (98%) and 2.84 g nitric acid (69%) in a ratio of 1 (H_2_SO_4_):2 (HNO_3_). Then, the concentrated acid (0.035 g) was diluted with distilled water (5 L) to an acid rain solution with a pH of 4 verified by the pH meter.

#### 2.1.3. Erosion Process

The erosion process was performed based on a previous study [26]. Twenty-eight grams of bitumen was evenly distributed on a glass container to a 0.85 mm thick film. The bitumen film was immersed in a 150 ml simulated acid rain at 25 °C for 7, 28, and 90 days. To avoid the light and temperature effects, bitumen samples were covered and sealed by a black plastic bag. After the erosion process, the solution was collected in a cup for subsequent testing, and the surface of the bitumen was washed with distilled water. To avoid the effect of moisture on the bitumen properties, the bitumen samples were dried in a fume hood for three days before the next steps.

#### 2.1.4. Abbreviations Used to Represent the Bitumen Samples

To simplify the description of bitumen samples under different conditions, the abbreviations of UT and AT were used to represent neat and TFOT aged bitumen, respectively. Acid-UT-7D, Acid-UT-28D, and Acid-UT-90D represent neat bitumen eroded in acid rain for 7, 28, and 90 days, respectively. Acid-AT-7D, Acid-AT-28D, and Acid-AT-90D indicate that aged bitumen has been eroded in acid rain for 7, 28, and 90 days, respectively.

### 2.2. Test Methods

#### 2.2.1. The Hydrogen Ions Analysis

The hydrogen ions of simulated acid rain with erosion time were measured by a pH 1000H meter. The pH value of the solution was tested at 20 °C with a resolution of 0.01. Each solution was tested twice, the average value of them was taken as the test result.

#### 2.2.2. Scanning Electron Microscopy (SEM) Analysis

The morphology testing of the bitumen with and without acid rain erosion was performed by scanning electron microscopy (SEM). The SEM images of the bitumen surface were captured at a magnitude of 200 times.

#### 2.2.3. Physical Properties

In this research, three physical parameters were evaluated; penetration at 25 °C, softening point, and complex viscosity at 60 °C, following EN 1426:2015 [22], EN 1427:2015 [23], and EN 13702:2018 [24], respectively. For the penetration test, one sample was tested for three measurements, and the average value of the three measurements was taken as the final penetration. Two samples were prepared for softening point and complex viscosity tests, and the average of the two measurements was taken as the test result.

#### 2.2.4. Attenuated Total Reflectance (ATR) Fourier Transform Infrared Radiation (FTIR) Spectroscopy Analysis

The functional groups of the bitumen were determined through an FTIR spectrometer equipped with a reflection diamond ATR accessory. The quantitative analyses of the functional groups were studied by the absorbance difference based on previous references [27,28]. Two replicates were for each sample, and the average absorbances of S=O, C=O, and C-O were regarded as the test result.

#### 2.2.5. Rheological Behaviour Analysis

The rheological behaviour of the bitumen was evaluated by a dynamic shear rheometer (DSR) according to EN 14770:2012 [29]. The low-temperature and high-temperature rheological properties of the bitumen were studied by conducting low-temperature creep and high-temperature creep, respectively. The test parameters for the different modes are described in Table 2.

## 3. Results and Discussion

### 3.1. The Hydrogen Ions of Simulated Acid Rain

The concentration of hydrogen ions of the acid solution would be influenced by the water content, while the water content would change during the erosion procedure due to the evaporation of moisture. The smaller the pH value, the higher the concentration of hydrogen ions of acid solution. Table 3 shows the volume loss of acid rain after a different number of days and the resultant pH value based on the volume loss. With increasing erosion time, the volume loss increased. Compared to aged bitumen, the acid rain after eroding neat bitumen had a more considerable volume loss under the same condition. This phenomenon can be interpreted by the stronger reaction between neat bitumen and acid rain. As the chemical reactions between bitumen and acid rain are mostly exothermic, and neat bitumen as a less stable substance can strongly react with acid rain than aged bitumen, this finally leads to more evaporation of the solution. According to the volume loss of acid solution, the pH value of acid rain will slightly decrease with erosion time. For instance, the pH value of acid rain after 7 days is 4; the acid rain after 28 days drops by 0.01 in pH value; and the acid rain after 90 days decreases by 0.07 (neat bitumen) and 0.03 (aged bitumen), respectively.

**Figure 1:**
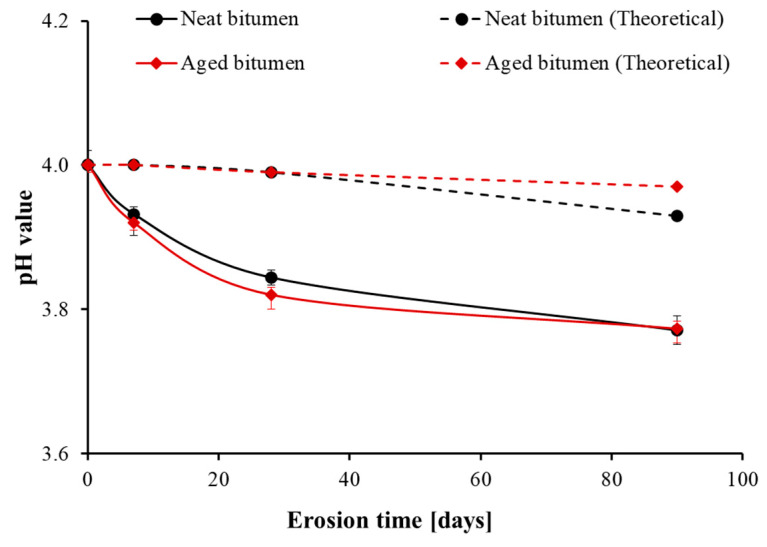
The pH value of simulated acid rain after eroding neat bitumen and aged bitumen.

Figure 1 shows the actual pH value of acid rain as a function of erosion time. It was found that the acid rain was influenced by the erosion process, reflected in the decrease in the pH value of the acid rain. The pH value of the acid rain decreased with increasing erosion time regardless of the bitumen, resulting in 3.76 (neat bitumen) and 3.77 (aged bitumen) after 90 days. Two slightly different developing trends were observed for neat bitumen and aged bitumen: the pH value of acid rain for neat bitumen decreased evenly, whereas the pH value of acid rain for aged bitumen decreased steeply before 28 days and gently after 28 days. However, the actual tested pH values of acid rain after the erosion process were far smaller than the theoretical pH value of the acid solution based on volume loss, and this difference increased with increasing erosion time. The above results indicate that the decreased pH value of the acid rain is mainly caused by the erosion process, and the influences of neat bitumen and aged bitumen on the pH value of the acid rain were similar. The reduction in pH value of residual acid rain might be originated from more dissolvable organic acid from bitumen to solution than that from solution to bitumen, which is an obvious difference compared to oxidative ageing.

### 3.2. Morphology of the Bitumen

**Figure 2:**
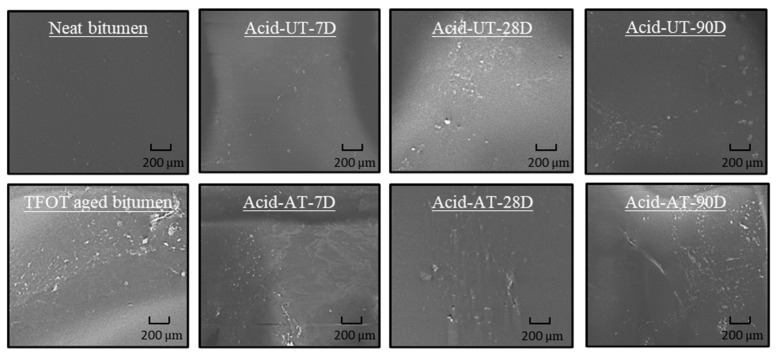
SEM images of the bitumen with and without acid rain erosion.

The SEM images of the bitumen with and without acid rain erosion are presented in Figure 2. There were fewer visible particles and lower apparent roughness on the surface of neat bitumen compared to aged bitumen for the same erosion time. This result indicates that TFOT ageing will lead to apparent roughness of the bitumen surface with few particles, and the erosion of acid rain would not change this trend. However, both neat bitumen and TFOT aged bitumen showed increased surface roughness with increasing erosion time in acid rain. This finding shows that acid rain might decompose the chemical bond of the bitumen, resulting in a rougher surface with particles.

### 3.3. The Physical Properties of the Bitumen

**Figure 3:**
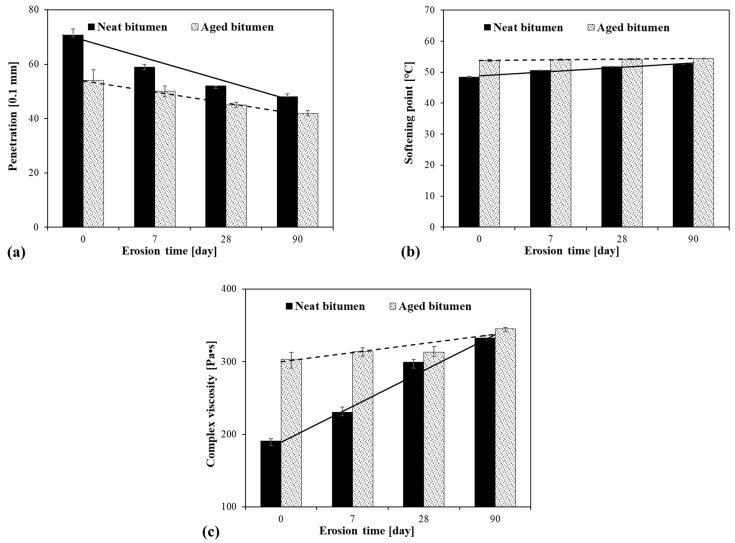
(**a**): Penetration; (**b**): softening point; (**c**): complex viscosity at 60 °C of the bitumen under different conditions versus erosion time.

Penetration, softening point, and complex viscosity are three typical physical parameters characterising the physical properties of bitumen [30]. The effect of acid rain on the physical properties of the bitumen is shown in Figure 3. It was found that there was a decrease in penetration with acid rain erosion and erosion time, a slight increase in softening point, and a sharp increase in complex viscosity. These results indicate that acid rain erosion makes bitumen stiffer, more stable, and better flow resistant. The degree of changes in the physical properties increased with increasing erosion time. It is worth to note that the physical properties of neat bitumen were more easily affected by erosion than aged bitumen since the increasing/decreasing slopes of the physical parameters with immersion time of neat bitumen were bigger than that of aged bitumen.

### 3.4. Chemical Structure of the Bitumen

There are three peaks worth noticing after acid rain erosion, namely, the carbonyl group, sulfoxide group, and carbonyl acid group located at 1700, 1030, and 1301 cm^−1^, respectively. Both carbonyl and sulfoxide groups are two oxygen-containing groups employed as two ageing level indicators of bitumen [31,32]. The carbonyl acid group in bitumen might be dissolved in acid rain; this action named the dissolution of carbonyl acid [33]. The information mentioned above for the three functional groups is shown in Table 4.

**Figure 4:**
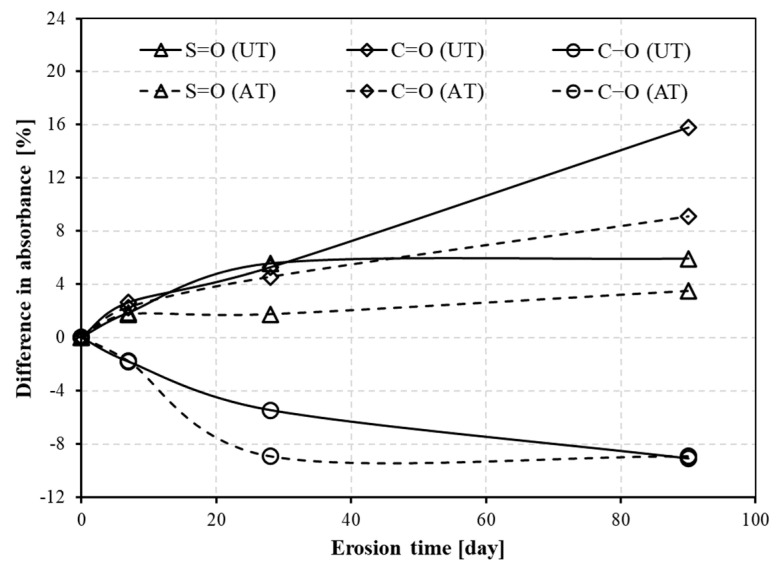
Difference in absorbance of neat bitumen and aged bitumen under acid rain erosion.

To quantitively analyse the development of chemical bonds of the bitumen during the erosion time, an absorbance difference is calculated at specific wavenumbers and plotted in Figure 4. As can be seen, both the carbonyl and sulfoxide group (C=O and S=O) increased after acid rain erosion, which implies that acid rain leads to ageing of the bitumen. The carbonyl acid group (C-O) decreased after acid rain erosion, which is completely different from oxidative ageing. This result indicates less of the carbonyl acid group within the bitumen. These outcomes will contribute to the hardening of the bitumen and more acid in the solution, resulting in higher stiffness and lower pH value of the solution, which is in line with the results from Section 3.1 and Section 3.3. The absolute values of the three functional groups increased with various speed after acid rain erosion. The C=O group of both neat bitumen and aged bitumen linearly increased with erosion time. The S=O group of both neat bitumen and aged bitumen slowly increased with erosion time and almost remained constant after 28 days of erosion, which changed minimally among the three groups. The absolute value of the C-O group of neat bitumen increased evenly with erosion time, whereas that of aged bitumen increased apparently before 28 days and kept changing slowly afterwards. The changing trend in the C-O group of neat bitumen and aged bitumen is similar to that in the pH value of acid rain. Neat bitumen resulted in a bigger absorbance difference in the C=O group compared to the S=O and C-O groups after acid rain erosion. Aged bitumen showed the most increase in the C-O group among the three groups. The above results indicate that neat bitumen is more easily aged than aged bitumen, while both neat bitumen and aged bitumen are dissolved similarly by acid rain. In other words, oxidation is the dominant action of neat bitumen during the erosion process, while dissolution is the typical action of aged bitumen.

### 3.5. The Rheological Properties of the Bitumen

**Figure 5:**
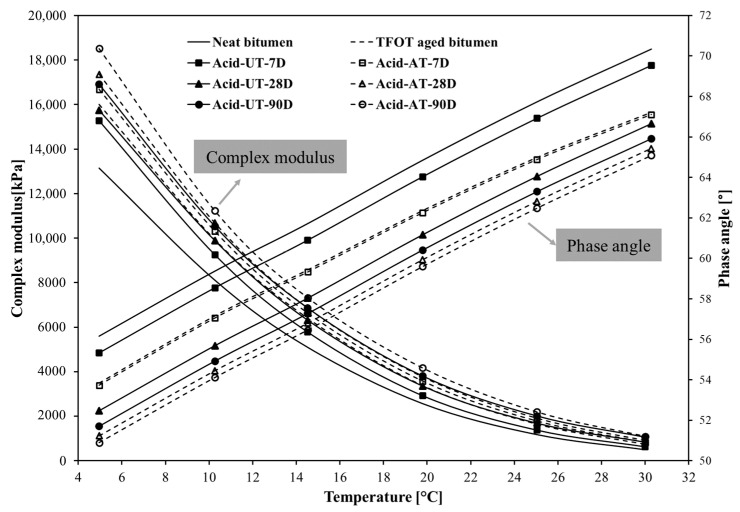
The complex modulus and phase angle of the bitumen under acid rain erosion at low temperature (5–30 °C).

**Figure 6:**
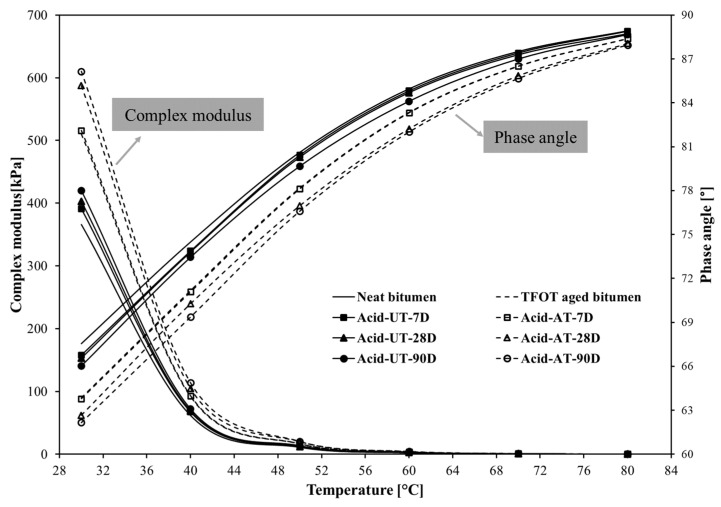
The complex modulus and phase angle of the bitumen at high temperature after acid rain exposure (30–80 °C).

The low-temperature rheological properties of the bitumen were analysed in the range 5–30 °C. The complex modulus (G*) of the bitumen under acid rain during the erosion process is plotted on the primary axis in Figure 5. Regarding neat bitumen, acid rain increased the complex modulus of the bitumen, and the complex modulus increased with increasing erosion time. Aged bitumen showed a similar trend as neat bitumen for acid rain erosion. These results indicate that acid rain increases the resistance to deformation of both neat and aged bitumen, and the deformation resistance continues to increase with increasing erosion time, which is in line with the results obtained from physical tests. However, better resistance to deformation at low temperature, an adverse effect on bitumen, is suspected of causing temperature cracks on asphalt pavements. Comparing neat bitumen and aged bitumen, the slope of complex modulus of neat bitumen (28.7%) with erosion time was slightly bigger than that of aged bitumen (15.6%). This result indicates that the deformation resistance of neat bitumen at low temperatures is more susceptible to acid rain than aged bitumen. The complex modulus of the TFOT aged bitumen is similar to that of Acid-UT-28D, which indicates that erosion in acid rain for 28 days has a similar impact on neat bitumen as TFOT ageing. The phase angle (δ) of the bitumen under acid rain during erosion time is plotted on the secondary axis in Figure 6. Both neat bitumen and aged bitumen were greatly influenced by acid rain. The phase angle decreased with the increasing erosion time. The results indicate that acid rain improves the elastic characteristic and deteriorates the viscous characteristic of bitumen, and the deterioration in viscous behaviour increases with increasing erosion time. However, the tendency of descending degree in the phase angle for neat bitumen was greater than that of aged bitumen, which indicates that neat bitumen is more vulnerable to acid rain erosion than aged bitumen. The phase angle of TFOT aged bitumen is bigger than for Acid-UT-28D and smaller than for Acid-UT-7D, which reveals that erosion in acid rain for 28 days has a similar impact on neat bitumen as TFOT ageing.

The high-temperature rheological properties of the bitumen were tested using high-temperature creep in the range 30–80 °C. The complex modulus (primary axis) and phase angle (secondary axis) of bitumen under acid rain over erosion time at high temperature is presented in Figure 6. It is observed that acid rain influenced the rheological properties of both neat bitumen and aged bitumen with different extents, resulting in increased complex modulus and decreased phase angle. The complex modulus and phase angle of neat bitumen continuously changed with the erosion time, while that of aged bitumen remained unchanged before 7 days and changed afterwards. Additionally, the changes in complex modulus and phase angle increased with the extension of the erosion time. These results indicate that acid rain leads to better deformation resistance and worse viscous characteristic of the bitumen, and the degree of change slightly increased with erosion time, which is consistent with the results at low temperature. Furthermore, the rate of increase (slope) in the complex modulus of neat bitumen (14.7%) caused by acid rain was smaller than that of aged bitumen (19.8%), and the rate of decrease in the phase angle of neat bitumen (2.3%) was also lower than that of aged bitumen (2.7%). These results indicate that acid rain has a more obvious effect on the deformation resistance and viscoelasticity for the aged bitumen at high temperature than neat bitumen. This phenomenon could be interpreted that the dissolution, the typical action of aged bitumen during the erosion process, makes the high-temperature rheological properties of bitumen more susceptible to acid rain.

**Figure 7:**
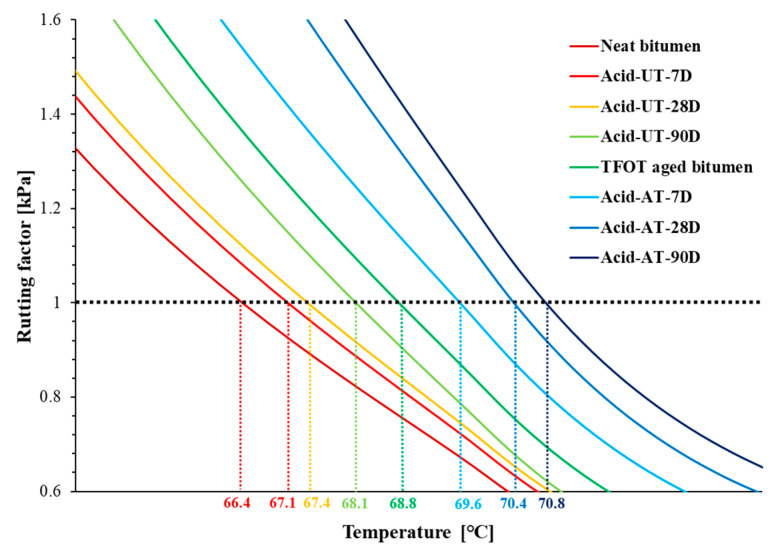
The rutting factor and failure temperature of bitumen.

The rutting factor G*/sinδ in the range 64–74 °C under the acid rain condition is shown in Figure 7. The acid rain led to an increased rutting factor of both neat bitumen and aged bitumen with erosion time, which indicates that acid rain erosion and longer erosion time resulted in better rutting resistance of bitumen. Based on the rutting factor at 1 kPa, the failure temperature was obtained for each sample recorded on the *X*-axis in Figure 7. The failure temperature indicating the critical temperature to rutting was also affected by acid rain and erosion time. It is found that both acid rain erosion and longer erosion time had a positive effect on the failure temperature of neat bitumen and aged bitumen. The degree of change in the failure temperature of neat bitumen (2.6%) was smaller than that of aged bitumen (2.9%), which reveals that rutting resistance of aged bitumen is more sensitive to acid rain compared to neat bitumen.

## 4. Conclusions

The interaction between bitumen and acid rain was investigated by analysing the morphology, physical properties, chemical structure, low-temperature and high-temperature rheological properties of bitumen, as well as the hydrogen ion concentration of the acid rain. The conclusions are summarised as follows:

After acid rain erosion, neat bitumen and aged bitumen have similar responses: more visible particles and apparent roughness of the bitumen surface regarding the morphology; decreased penetration, increased softening point, and complex viscosity in terms of physical properties; more C=O and S=O groups and fewer C-O groups regarding chemical structure; increased complex modulus, rutting factor, and failure temperature, and decreased phase angle concerning rheological properties. The erosion process led to more hydrogen ions in the acid rain. In addition, the changes in the above properties were positively related to erosion time. The above conclusions demonstrate that the acid rain has a similar effect on the physical and rheological properties of bitumen compared with the oxidative ageing, while different chemical responses of bitumen were found for acid rain compared to the oxidative ageing.

Some differences were found in the response of neat bitumen and aged bitumen to acid rain. The oxidative functional groups (C=O and S=O), physical and low-temperature properties of neat bitumen were more vulnerable to acid rain than for aged bitumen, while the high-temperature rheological properties of aged bitumen were more susceptible to acid rain than neat bitumen. The effect of the erosion process on hydrogen ions of acid rain and the carbonyl acid group of neat bitumen was similar to that of aged bitumen.

The deterioration mechanism could be that both oxidation and dissolution occurred during the erosion process, resulting in ageing of the bitumen and eroding damage on the bitumen. The outcomes of oxidation were mainly increased C=O and S=O groups, and the dissolution typically resulted in fewer C-O groups of the bitumen and higher concentration of hydrogen ions within the acid rain. Both the oxidation and the dissolution caused changes in the morphological, physical, and rheological properties of the bitumen.

The oxidation is the dominant action of neat bitumen during the erosion process, while the dissolution reaction is the typical action of the aged bitumen. The different typical reactions of neat bitumen and aged bitumen eventually lead to different responses to acid rain of neat bitumen and aged bitumen.

The presented results are only valid for neat bitumen and aged bitumen in existing road surfaces. It is meaningful to conduct further tests on different kinds of bitumen for finding the best type of bitumen that resists acid rain erosion.

## Figures and Tables

**Table 1:** The physical and chemical properties of the bitumen.

Properties		Unit	Neat Bitumen	Aged Bitumen	Test Standard
penetration (25 °C)		0.1 mm	71	54	EN 1426:2015 [22]
softening point		°C	48.4	53.8	EN 1427:2015 [23]
viscosity (60 °C)		Pa∙s	191	303	EN 13702:2018 [24]
absorbance(FTIR)	S=O	1030 cm^−1^	0.054	0.057	FTIR analysis [25]
	C=O	1700 cm^−1^	0.019	0.022	
	C-O	1301 cm^−1^	0.055	0.055	

**Table 2:** Test parameters of the two test modes.

Mode	Low-Temperature Creep	High-Temperature Creep
sweep frequency (rad/s)	10	10
temperature range (°C)	5–30	30–80
temperature interval (°C)	5	10
diameter of the plate (mm)	8	25
sample thickness (mm)	2	1

**Table 3:** Volume loss and calculated theoretical pH value of acid rain under different conditions.

Condition	Acid-UT-7D	Acid-AT-7D	Acid-UT-28D	Acid-AT-28D	Acid-UT-90D	Acid-AT-90D
volume loss (mL)	0.02	0.02	3.67	2.35	22.36	11.50
theoretical pH	4.00	4.00	3.99	3.99	3.93	3.97

**Table 4:** Three typical chemical groups of the bitumen under acid rain erosion.

Peak Position	Functional Group	Description	Indication
1030 cm^−1^	S=O	Sulfoxide group	Oxidation
1700 cm^−1^	C=O	Carbonyl group	
1301 cm^−1^	C-O	Acetate ester/Carbonyl acid	Dissolution

## Data Availability

Data available on request from the corresponding author.
